# Wetlands harbor lactic acid-driven chain elongators

**DOI:** 10.1128/spectrum.02105-23

**Published:** 2023-12-12

**Authors:** Pieter Candry, Zachary Flinkstrom, Mari-Karoliina Henriikka Winkler

**Affiliations:** 1 Civil and Environmental Engineering, University of Washington, Seattle, Washington, USA; Dominican University New York, Orangeburg, New York, USA

**Keywords:** wetland, carbon cycle, chain elongation, medium-chain carboxylic acids

## Abstract

Wetlands are globally significant carbon storage hotspots. Recent research has suggested that microbially derived metabolites may contribute to soil organic matter formation. Identifying pathways driving the formation of such metabolites is critical to understand the global impact of wetland carbon cycling. Here, we evaluate the presence of chain-elongating organisms converting two to three carbon compounds (i.e., lactic and acetic acid) to medium-chain carboxylic acids (MCCA; i.e., six-carbon caproic acid) in wetland soils. We demonstrate the enrichment of a lactic acid-driven chain-elongating community from wetland soils producing a mixture of butyric and caproic acid. The enriched community was dominated by Clostridiaceae, Ruminococcaceae, and Lachnospiraceae, three families with known chain elongators. Amplicon sequencing identified three Ruminococcaceae and one Clostridiaceae zero-radius OTU (zOTU) that were (i) present in the soil, (ii) enriched over 1% relative abundance in the bioreactor, and (iii) were closely related to known chain elongators. Moreover, close relatives of the three Ruminococcaceae zOTU were also observed in several other wetland microbiomes. From this observation, we conclude that close relatives of known chain elongators, potentially capable of lactic acid-driven MCCA production themselves, are present in wetland soils. This observation may have implications for our understanding of carbon cycling and storage in wetland ecosystems.

**IMPORTANCE:**

Wetlands are globally significant carbon cycling hotspots that both sequester large amounts of CO_2_ as soil carbon as well as emit a third of all CH_4_ globally. Their outsized role in the global carbon cycle makes it critical to understand microbial processes contributing to carbon breakdown and storage in these ecosystems. Here, we confirm the presence of chain-elongating organisms in freshwater wetland soils. These organisms take small carbon compounds formed during the breakdown of biomass and turn them into larger compounds (six to eight carbon organic acids) that may potentially contribute to the formation of soil organic matter and long-term carbon storage. Moreover, we find that these chain-elongating organisms may be widely distributed in wetlands globally. Future work should identify these organisms’ contribution to carbon cycling in wetlands and the potential role of the products they form in carbon sequestration in wetlands.

## OBSERVATION

Wetlands store 20%–30% of global soil carbon and emit approximately one-third of CH_4_ emissions within just 5%–6% of land area (1, 2). Microbial breakdown of plant-fixed carbon eventually either releases this carbon as greenhouse gases (CO_2_, CH_4_) or transforms it into soil organic matter (SOM). While hydrolysis inhibition in the absence of O_2_ has been proposed as a key driver behind SOM persistence and long-term carbon storage, microbially derived metabolites may also contribute to stable SOM formation (3 – 5). Understanding novel carbon cycling pathways in wetlands is critical to map microbially produced SOM fractions.

Microbial chain elongation anaerobically converts two to four carbon compounds (e.g., ethanol, acetic acid, lactic acid, etc.) into six to eight carbon medium-chain carboxylic acids (MCCA, e.g., six-carbon caproic acid) (6) whose aliphatic nature and potential mineral-organic interactions might contribute to SOM formation (7, 8). While research on chain elongators has mostly focused on biotechnological applications, their presence in and contribution to environmental systems, specifically wetlands, remain understudied and poorly understood.

Chain elongators have been observed in environmental settings. For instance, *Clostridium kluyveri*—an ethanol-consuming chain elongator—was isolated from canal mud (9), while two recent studies enriched ethanol-elongating mixed communities from environmental samples including soils and animal feces (10, 11), and another study incidentally observed chain elongation in H_2_-supplied soil incubations (12). Despite their potential significance, the presence of lactic acid-consuming chain elongators in wetlands—or other terrestrial environments—has not yet been investigated.

A lactic acid-driven chain-elongating community was enriched from urban lacustrine wetland soil (47° 38′ 31.12′′ N, 122° 17′ 47.01′′ W, Table S1). Soil was inoculated (1% wt/vol) in a continuously stirred tank reactor (CSTR) with online pH control (pH 5.5) fed with a synthetic lactic and acetic acid medium (100 and 25 mM, respectively). Effluent was analyzed for metabolites and community composition (16S rRNA gene V4-V5 region amplicon sequencing). Additional methodological details on incubation conditions, chemical analyses, DNA extraction, PCR, and bioinformatic analyses can be found in supplemental materials (Section S1).

**Fig 1:**
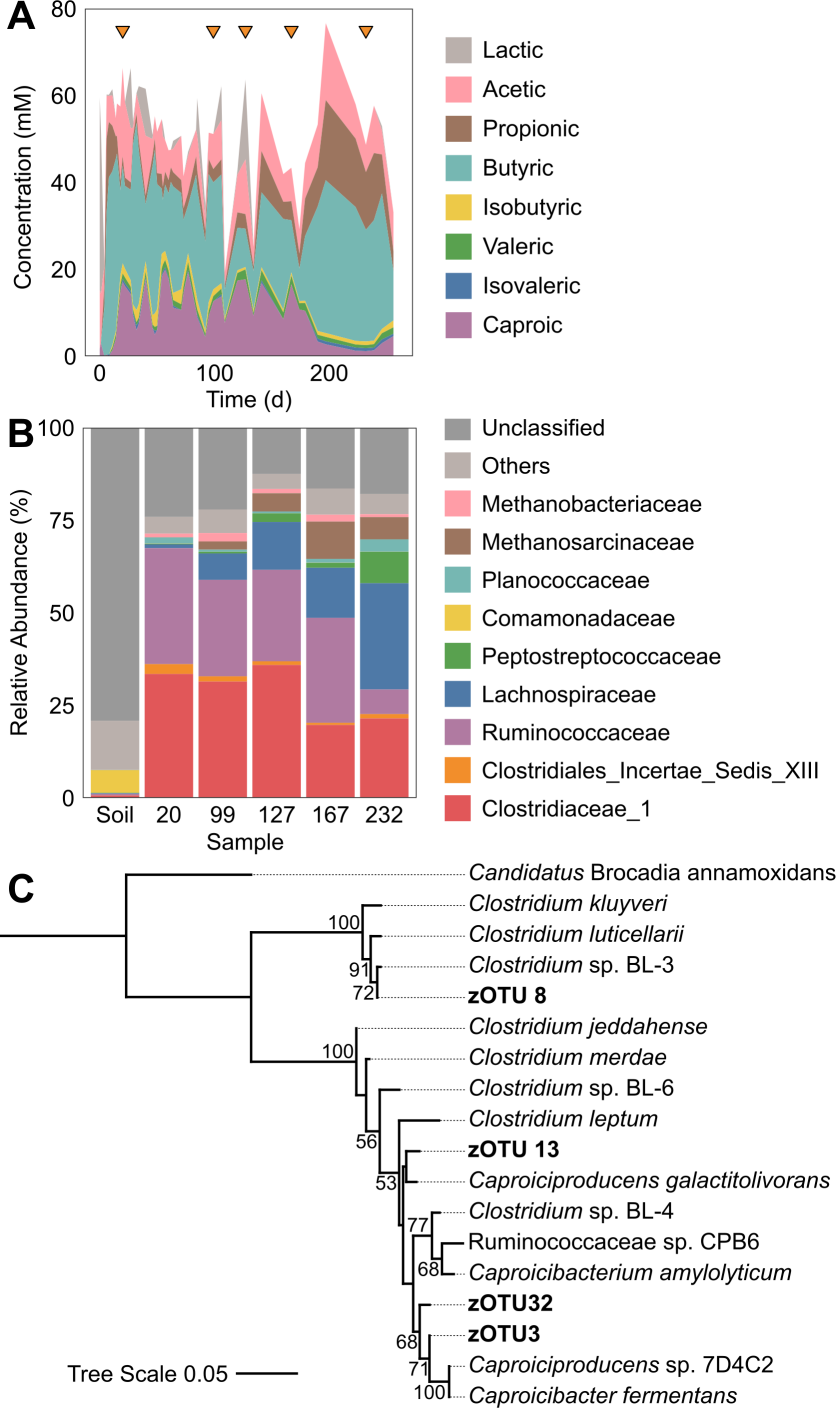
Enrichment of a lactic acid-driven chain elongation community from wetland soil. Panel (A) shows CSTR effluent product profiles with orange triangles indicating days sampled for community characterization. Panel (B) shows the relative abundance of community members classified at the family level. Panel (C) shows a phylogenetic tree of the V4-V5 region of the 16S rRNA gene for zOTU of interest along with relevant relatives. zOTU of interest (i) were detected in the wetland soil inoculum, (ii) had a relative abundance of at least 1% at any time during reactor operation, and (iii) are related to known chain elongators. Bootstrap values greater than or equal to 50% are shown as percentages at each node. Scale indicates substitutions per nucleotide position.

Chain elongation was observed almost immediately after startup (Fig. 1A). Butyric and caproic acid concentrations fluctuated throughout the enrichment, resulting in variable effluent concentrations (butyric acid: 21.94 ± 11.26 mM; caproic acid: 9.36 ± 6.21 mM) and a product profile that was alternately dominated on electron-equivalent basis by caproic acid or butyric acid (Fig. S1). Overall, even-chain products (i.e., butyric, caproic acid) consistently dominated the product profile (74.0% ± 15.1%), while odd-chain (i.e., propionic, valeric acid; 10.7% ± 7.1%) and branched-chain acids (i.e., iso-butyric, iso-valeric acid; 3.77% ± 2.3%) were minor by-products. Chain elongation was inferred to account for 48.4% ± 5.1% of metabolism from day 20 onward (Fig. S2). The production of higher alcohols and off-gas was not monitored as MCCA were the primary focus of this study. These results confirm lactic acid-driven chain elongation communities can be enriched from wetland ecosystems.

Amplicon sequencing was used to analyze community composition over the course of the enrichment. Broadly, three families accounted for over half of the community throughout the enrichment (Fig. 1B): (i) Clostridiaceae (20%–35% relative abundance), (ii) Ruminococcaceae (gradual decrease from 31.3% to 6.7% relative abundance), and (iii) Lachnospiraceae (gradual increase from 1.0% to 28.7% relative abundance). While all three families harbor known chain elongators (13), each of these families contains a wide range of functional diversity beyond chain elongation.

To evaluate which chain elongators may be present in wetlands, zOTU of interest were selected based on (i) classification at the family level, (ii) presence in the inoculum, (iii) presence at a relative abundance of at least 1% during enrichment, and (iv) phylogenetic relation to known chain elongators. These criteria identified three Ruminococcaceae (zOTU 3, 13, and 32) and one Clostridiaceae (zOTU 8) of interest (Fig. 1C). Of the three Ruminococcaceae zOTU, zOTU 3 had the highest relative abundance in the soil inoculum (0.17%) and throughout most of the enrichment (up to 15.4%; Fig. S3). This zOTU was most closely related to *Caproiciproducens* sp. 7D4C2 and *Caproicibacter fermentans* [hexoses to caproic acid (14, 15)]. zOTU32 (inoculum abundance: 0.008%) was closely related to zOTU3 and its representative isolates but maintained lower relative abundances throughout enrichment (0.03%–1.28%). zOTU 13 (inoculum abundance: 0.03%) was present at low abundances in the enrichment (0.66%–3.72%) and most closely related to *Caproiciproducens galactitolivorans* [sugar alcohols to caproic acid (16)]. Last, the Clostridiaceae zOTU (inoculum abundance: 0.05%) was closely related to *Clostridium luticellarii* [methanol to (iso)butyric and caproic acid (17)] and *Clostridium* sp. BL-3 [lactic acid to (iso)butyric acid (18)]. This analysis shows likely chain elongators were present in the original wetland soil.

We evaluated the presence of these putative chain elongators in other environmental and engineered systems by searching the zOTU in public sequencing data (S.1.6.). All zOTU were found in other systems (Fig. S4 and S5; Table S3). The four zOTU were present in 33%–86% of all bioreactor or enrichment samples (*n* = 275, median abundances of 0.0022%–40.5209%), while detection in wetland sites was sparser with zOTU 3, 13, and 32 detected in 1.6%–7.1% of samples (*n* = 127, median abundances of 0%–0.0141%). zOTU 8 was not detected in any wetlands besides the site studied here. Notably, saline, sulfate-rich soils contained none of the four zOTU, potentially due to substrate competition between sulfate reducers and chain elongators in these settings (13).

The data presented here indicate lactic acid chain-elongating communities can be enriched from wetland soils, and putative chain elongators were present in a range of wetland soils.

The functionality of zOTU was inferred from abundances in the enrichment and their closest phylogenetic relatives. However, close relatives and even different strains within a single species may exhibit divergent properties. Moreover, predicting chain elongation potential from full genomes remains a challenge, which is further complicated by some isolates producing MCCA only under certain conditions (15, 19, 20). Given these limitations, we conclude that close relatives of known chain elongators, potentially capable of lactic acid-driven MCCA production themselves, were found in wetland soils. It should be highlighted that this is the first report of chain elongators in wetland ecosystems as well as the first report of lactic acid chain elongators in natural environments.

The presence of lactic acid chain elongators in wetlands may have implications for our understanding of wetland carbon cycling. Short-chain carboxylic acids (e.g., acetic, propionic, and lactic acid) are typically converted to CH_4_ under anaerobic conditions. If chain elongators are present and active in wetlands, they may divert carbon away from CH_4_ toward MCCA, potentially sequestering carbon and altering the system’s net greenhouse gas flux. While we show their presence and potential role, open questions remain on which environmental conditions favor chain elongators in wetlands and to what extent they alter the microbial cross-feeding network in the ecosystem (11, 13). Chain elongators have a competitive advantage over methanogenic consortia in spatiotemporal niches with elevated H_2_ partial pressures (21). Such temporal niches may occur during periods of flooding or high organic matter influx (22, 23), or in spatial niches allowing longer-term proliferation of chain elongators. In parallel, interactions between MCCA and SOM (7, 8) could extend the retention time of carbon in the system, providing a potential mechanism for chain elongators to simultaneously reduce CH_4_ flux by outcompeting methanogens as well as contribute to longer-term carbon storage. These hypotheses, however, remain to be confirmed with experimental data.

This study presents the enrichment of a lactic acid-driven chain elongation community from wetland soils and identifies close relatives of known chain elongators that were present in the original soil as well as other wetland soils. This observation may have implications for our understanding of carbon cycling and storage in wetland ecosystems.

## Data Availability

Raw amplicon sequencing data are available from the NCBI SRA under BioProject accession PRJNA973099.

## SUPPLEMENTAL MATERIAL

Supplemental material spectrum.02105-23-s0001.pdf Additional information on methodology and additional supporting data.

ASM does not own the copyrights to Supplemental Material that may be linked to, or accessed through, an article. The authors have granted ASM a non-exclusive, world-wide license to publish the Supplemental Material files. Please contact the corresponding author directly for reuse.
