# Supplementary material for: Wetlands harbor lactic acid-driven chain elongators
Source: Microbiol Spectr. 2023 Dec 12;12(1):e02105-23. doi: 10.1128/spectrum.02105-23 (PMC10783096; doi:10.1128/spectrum.02105-23)
Supplement: Supplemental material — Additional information on methodology and additional supporting data. [file spectrum.02105-23-s0001.pdf]

Supplemental materials to: Wetlands harbor lactic acid-driven chain elongators

Pieter Candry[1,#,*], Zachary Flinkstrom[1], Mari Karoliina-Henriikka Winkler[1]

[1] Civil and Environmental Engineering, University of Washington, 201 More Hall, Box 352700, Seattle, WA 98195-2700, USA

[#] Correspondence to: Pieter Candry, Civil and Environmental Engineering, University of Washington, 201 More Hall, Box 352700, Seattle, WA 98195-2700, USA; phone: +1-206-556-8282; E-mail: pcandry@uw.edu

* Current Address: Laboratory of Systems and Synthetic Biology, Wageningen University & Research. 6708 WE, Wageningen, The Netherlands; E-mail: pieter.candry@wur.nl

S.1. Materials & Methods

S.1.1. Inoculum

Chain elongating communities were enriched from a hydric organic-rich peat/muck type soil taken from an urban lacustrine wetland (University of Washington Arboretum, Seattle, WA, USA; 47° 38' 31.12'' N, 122° 17' 47.01'' W). Samples were taken using a 2"x4" soil corer (AMS, American Falls, ID, USA) driven down 60 cm into the soil. Cores were transported to the lab on blue ice and stored at 4°C until the start of the experiment.

Table S.1. Geochemical characteristics of the wetland sampling site based on porewater. Samples were taken using a porewater dialysis sampler covering the top 60 cm of soil in 3 cm increments.

Analyte	Range
pH	7.2-7.9
H_2S	Not detected – 132 µM
SO_4^-	3.40 – 465 µM
$NH_3 + NH_4^+$	15.4 – 483 µM
NO_2^-	Not detected
NO_3^-	Not detected – 49.9 µM
Lactate	Not detected – 8.29 µM
Acetate	Not detected – 2090 µM
Propionate	Not detected – 329 µM
Formate	Not detected – 8.47 µM
Butyrate	Not detected – 147 µM

S.1.2. Reactor operation

Chain elongating communities were enriched in a continuously stirred tank reactor (CSTR). The CSTR was inoculated with 1% (w/v) wet soil in a synthetic lactate-acetate medium modified from previous reports (1).

Specifically, the medium contained (in 1000 mL MQ water); 28.5 mL Na-Lactate as 60% syrup (100 mM), 4.1 g Na-Acetate (25 mM), 0.436 g $MgCl_2 \cdot 6H_2O$, 0.147 g $CaCl_2 \cdot 2H_2O$, 0.29 g $NaH_2PO_4 \cdot 2H_2O$, 6.7 g KCl, 1.91 g NH_4Cl, 1.0 g yeast extract. This basal medium was autoclaved at 121°C for 30 minutes, after which trace elements were added (per L medium):

10 mL SL-10 trace element solution, 10 mL selenate-tungstate solution, and 0.1 mL 7-vitamin solution. The SL-10 trace element solution consisted of (in 1000 mL MQ water): 10 mL 7.7M HCl, 1.5 g $FeCl_2.4H_2O$, 0.07 g $ZnCl_2$, 0.15 g $MnCl_2.4H_2O$, 0.006 g H_3BO_3, 0.19 g $CoCl_2.6H_2O$, 0.002 g $CuCl_2.2H_2O$, 0.024 g $NiCl_2.6H_2O$, 0.036 g $Na_2MoO_4.4H_2O$. The Se-W solution contained (in 1000 mL MQ water): 0.5 g NaOH, 0.003 g $Na_2SeO_3.5H_2O$, 0.004 g $Na_2WO_4.2H_2O$. The 7-vitamin solution consisted of (in 100 mL MQ water): 0.1 g vitamin B12, 0.08 g p-aminobenzoic acid, 0.02 g D(+)-Biotin, 0.2 g nicotinic acid, 0.1 g Ca-pantothenate, 0.3 g pyridoxine hydrochloride, 0.2 g thiamine-HCl.$2H_2O$.

The CSTR was operated with a hydraulic retention time (HRT) of 4 days and temperature controlled at 35°C. The pH was controlled to 5.5 with 1M NaOH or 1M HCl using a cRIO controller system (National Instruments, Austin, TX).

S.1.3. Analytical methods

Samples were taken from the effluent line of the reactor and analyzed immediately for optical density at 600 nm (OD600) with a Spectronic 200 spectrophotometer (Thermo Fisher Scientific, Waltham, MA). Part of the sample was diluted 1:1 with 20 mM KOH followed by filtering (0.20 µm) and storage at -20°C for later organic acid analysis. The remaining volume of unprocessed sample was stored directly at -20°C for later DNA extraction and community analysis.

Organic acids (C2-C6, including C4 & C5 isoforms) were analyzed with ion chromatography (IC) on a Dionex ICS 5000+ equipped with an IonPac AS11HC analytical and AS11 guard column at 30°C. Eluent consisted of generated KOH (Dionex EG-5 & Dionex EGCIII KOH, Thermo Fisher Scientific) at a flow rate of 1.5 $mL \cdot min^{-1}$. An eluent concentration gradient of 1-60 mM KOH was applied by first running 7 minutes at 1 mM, increasing at (i) 1.56 $mM \cdot min^{-1}$ for 9 minutes, then (ii) 1.67 $mM \cdot min^{-1}$ for 9 minutes, and eventually (iii) 3.75 $mM \cdot min^{-1}$ for 8 minutes to 60 mM, where concentration was held for 90 seconds. Concentration

was then decreased as a step function back to 1 mM KOH for 2 minutes before the end of the run. Component concentrations were externally calibrated (0.5-25 mg·L^{-1}) and validated every 10 samples.

Higher alcohol and gas (i.e., CH_4, CO_2, H_2) production were not analyzed in these experiments, as the focus of the study was on identifying the presence of lactic acid-driven chain elongation in wetland ecosystems.

S.1.4. Calculations

To calculate electron equivalents of products, molar product concentrations were multiplied by the number of electrons released during oxidation, i.e. acetate (8 mol e^{-}·mol^{-1}), propionate (14 mol e^{-}·mol^{-1}), (iso)-butyrate (20 mol e^{-}·mol^{-1}), (iso)-valerate (26 mol e^{-}·mol^{-1}), caproate (32 mol e^{-}·mol^{-1}). Product specificities were calculated as the fraction of the total electron pool in the effluent represented by any product. Pathway contributions were calculated according to previously published approaches (1). Briefly, from the biochemical understanding of metabolic pathways producing carboxylic acids, it is possible to infer key intermediates involved for each product. This knowledge is condensed in the following rules: (i) each odd-chain carboxylic acid requires one molecule of lactic acid to have gone over propionyl-CoA, (ii) each product with four or more carbon atoms requires lactic acid to have been used to produce acetoacetyl-CoA, or more generally, 2-ketoacyl-CoA, and (iii) each even-chain product requires one lactic acid to be oxidized to acetyl-CoA to start the cycle (Table S.1.).

Table S.2. Contribution of each acid towards each of the intermediates used in different metabolic pathways. The stoichiometric lactic acid consumption is the sum of lactic acid attributed to each intermediate (i.e. propionyl-CoA, 2-ketoacyl-CoA or acetyl-CoA).

Acid	Propionyl-CoA ($mol \cdot mol^{-1}$)	2-Ketoacyl-CoA ($mol \cdot mol^{-1}$)	Acetyl-CoA ($mol \cdot mol^{-1}$)	Total stoichiometric lactic acid consumption ($mol \cdot mol^{-1}$)
Acetic	-	-	1	1
Propionic	1	-	-	1
Butyric	-	1	1	2
Valeric	1	1	-	2
Caproic	-	2	1	3
Heptanoic	1	2	-	3
Octanoic	-	3	1	4

S.1.5. Community characterization

DNA was extracted from selected bioreactor samples as well as from the initial soil inoculum using the DNeasy PowerSoil Pro kit (Qiagen, Hilden, Germany) according to the manufacturer's protocol. The concentration of DNA was quantified using the Qubit™ dsDNA High Sensitivity Assay (Thermo Fisher Scientific, Waltham, MA, USA) and normalized with nuclease-free water to 2.5 ng/µL. The V4-V5 region of the 16S rRNA gene was PCR amplified by mixing the following ingredients to a final volume of 25 µL : 12.5 µL of LongAmp® Taq 2x master mix (New England Biolabs, Ipswich, MA, USA), 1 µL of 10µM stocks of 515F and 926R primers (2) with partial Illumina adapter sequences, 2 µL of DNA sample, and 8.5 µL of nuclease-free water. Samples were amplified according to the following process: denaturation at 94 °C for 30 s, 50 °C for 45 s, and 65 °C for 30 s for 30 cycles, with a final extension at 65 °C for 10 min. Agarose gel electrophoresis was performed to confirm the size and purity of

PCR products. PCR products were purified using the DNA Clean & Concentrator kit (Zymo Research, Irvine, CA, USA) and sent to Genewiz (South Plainfield, NJ, USA) for Amplicon-EZ Illumina sequencing.

S.1.6. Bioinformatic analyses

Demultiplexed and adapter-trimmed reads were analyzed with USEARCH v11 (3). Paired-end reads were merged using fastq_mergepairs, primer sequences were removed using fastx_truncate, and reads were quality filtered using fastq_filter with the fastq_maxee option set to 1.0 to remove reads with greater than one expected error. Unique reads and their abundances were computed using fastx_uniques followed by denoising and chimera removal using the unoise3 command (4). Reads were mapped to the resulting zero-radius OTUs using the otutab command. The taxonomy of the zero-radius OTUs (zOTU) was assigned using the nbc_tax command to search against the RDP 16S database v18 (5).

Specific zOTU of interest were filtered based on the following requirements: (i) zOTU were classified at the family level, (ii) zOTU were detected in the original soil inoculum, and (iii) zOTU were present at a relative abundance of at least 1% in any timepoint during enrichment.

A maximum-likelihood phylogenetic tree was built from V4-V5 regions of the 16S rRNA genes of zOTU of interest along with relevant close relatives. Sequences were aligned in MEGA X (6) using the Multiple Sequence Comparison by Log-Expectation (MUSCLE) alignment tool. A phylogenetic tree was constructed with the Maximum Likelihood algorithm, using the Tamura-Nei model (7) to calculate phylogenetic distances, and performing bootstrap analysis (n=1000) to assess the reliability of each branch. The phylogenetic tree was then visualized with the online Interactive Tree of Life webtool (8).

Publicly available 16S rRNA amplicon sequencing libraries were retrieved from the NCBI SRA using the sra-tools fasterq-dump command. Paired-end read libraries were first

merged using the usearch fastq-mergepairs command. Then reads were then searched for zOTU of interest using the usearch_local command with a minimum identity of 97% and a minimum alignment length of 200 bp. Relative abundance was computed using the number of hits to each zOTU of interest divided by the number of reads searched multiplied by 100.

S.2. Results

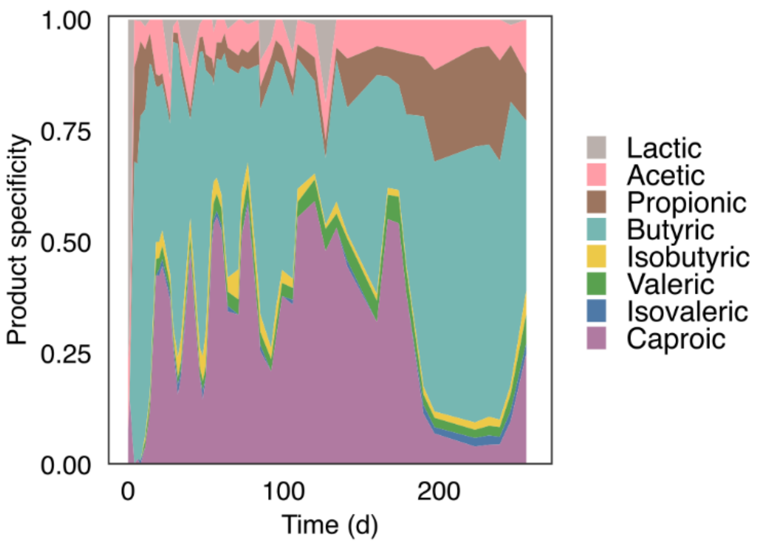

Figure S.1. Product specificity of a chain elongation community enriched from wetland soil. Specificity of the effluent product profile was calculated on electron basis.

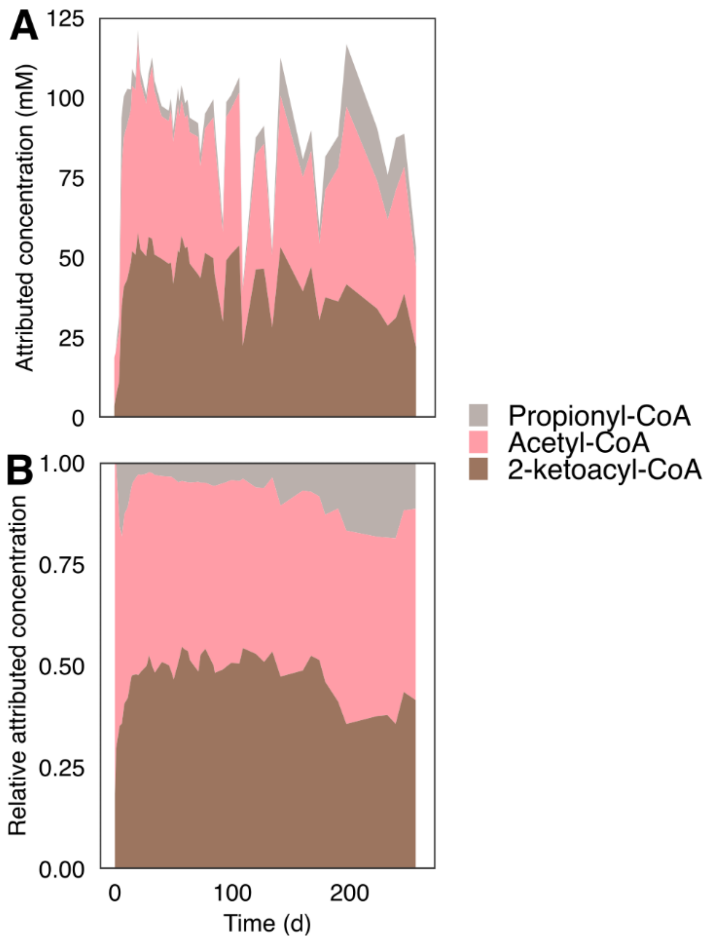

Figure S.2. Pathway distribution of a chain elongating consortium enriched from wetland soil. Pathway contributions were calculated according to Candry et al. (1). Panel A shows attributed concentration in molar concentrations, Panel B shows the same but as relative contributions on molar basis.

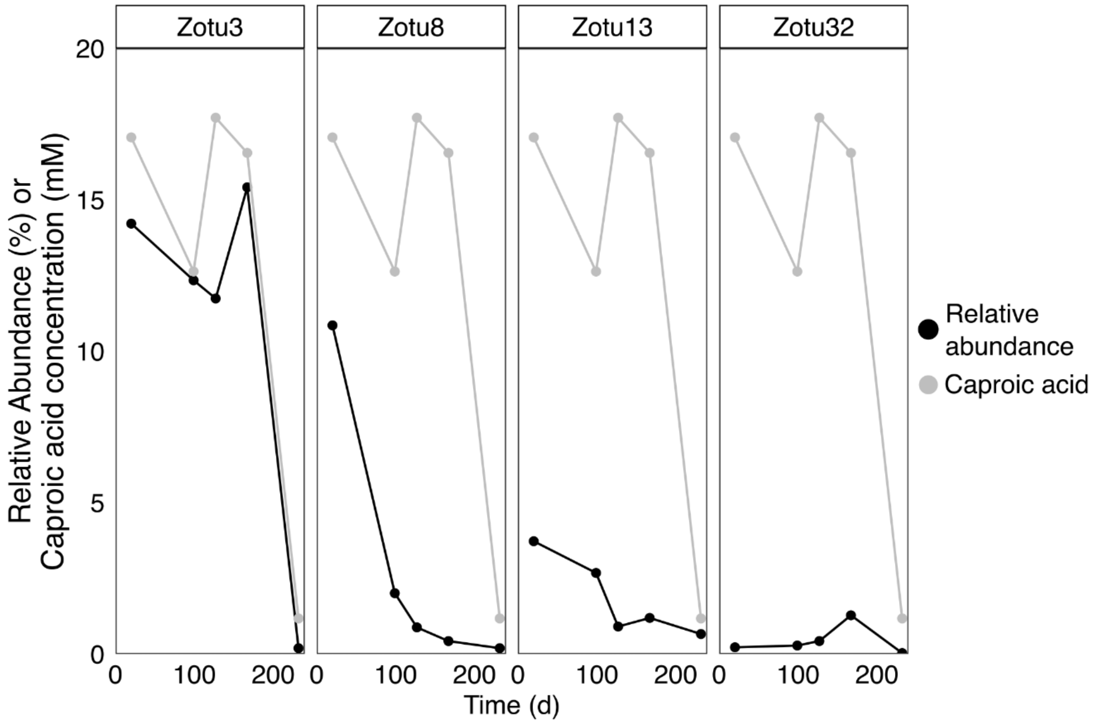

Figure S.3. Comparison or relative abundances of zOTU of interest and caproic acid concentrations.

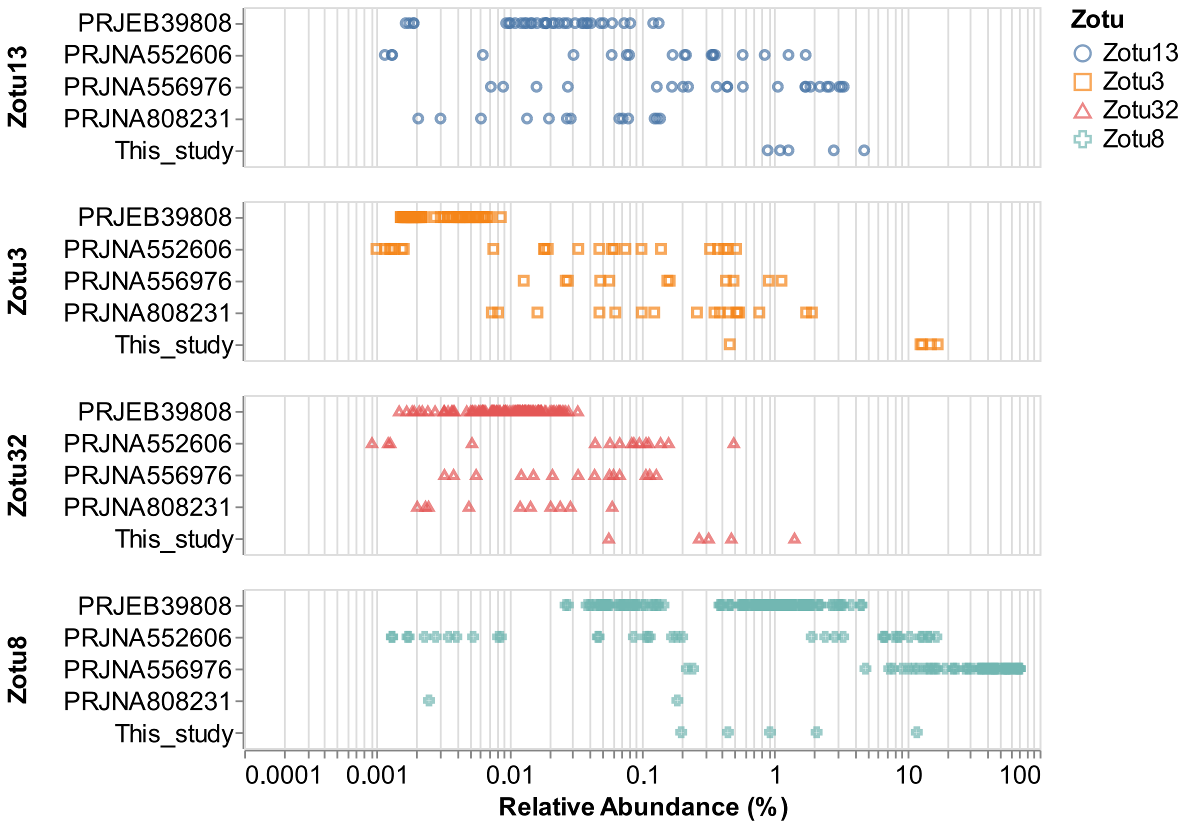

Figure S.4. Relative abundance of zOTU of interest in publicly available 16S rRNA amplicon sequencing data from ethanol or lactate-driven chain elongation bioreactor or enrichment studies. Undetected samples not shown.

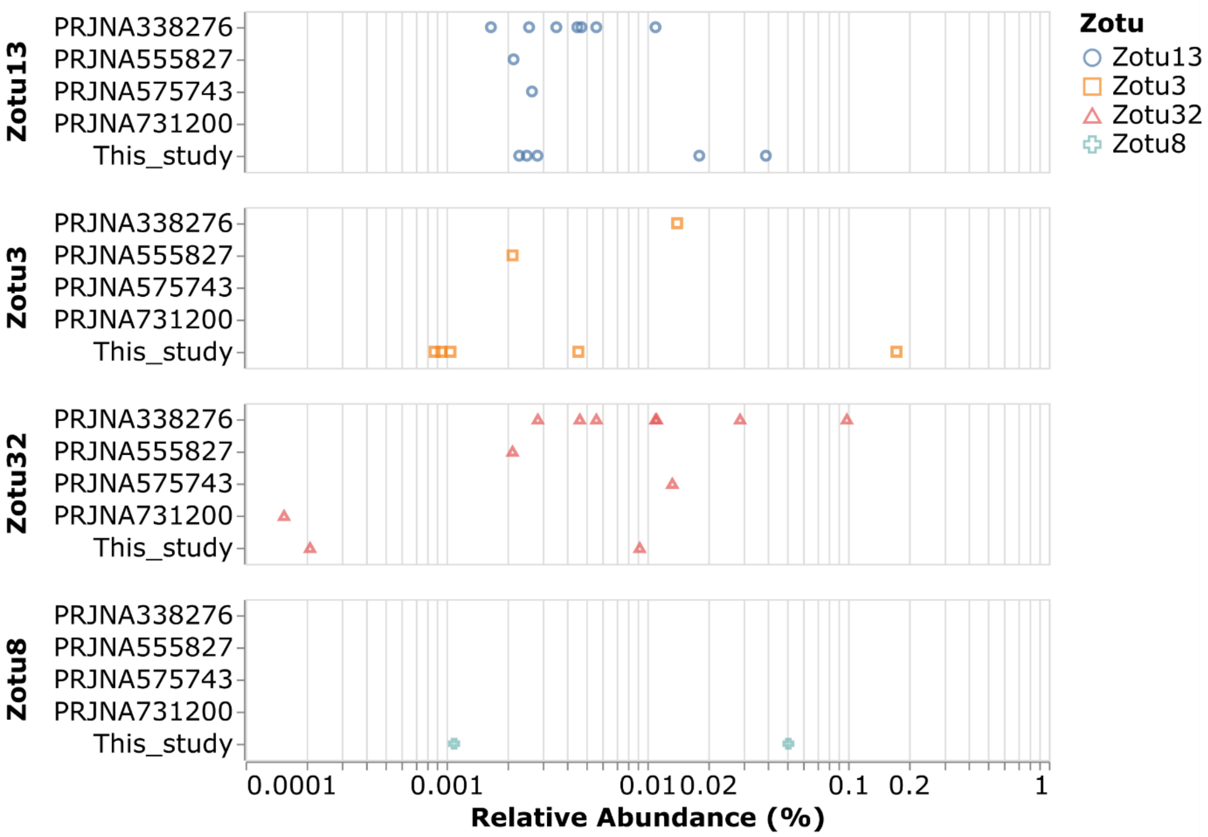

Figure S.5. Relative abundance of zOTU of interest in publicly available 16S rRNA amplicon sequencing data from wetlands. Undetected samples not shown.

Table S.3. Prevalence and median abundance of zOTU of interest in publicly available 16S rRNA amplicon sequencing libraries. Prevalence is defined as the percent of samples with a relative abundance of greater then 0.001% and the median is calculated based on only the samples with relative abundances greater than 0.001%.

BioProject	Study	Environment	Sample count	Zotu3 prevalence (%)	Zotu8 prevalence (%)	Zotu13 prevalence (%)	Zotu32 prevalence (%)	Zotu3 median abundance (%)	Zotu8 median abundance (%)	Zotu13 median abundance (%)	Zotu32 median abundance (%)
**PRJNA973099**	Lactate driven chain elongation in wetlands (This study)	Bioreactor	5	100.0	100.0	100.0	100.0	12.9935	0.9234	1.2733	0.3183
**PRJEB39808**	Increasing pH to shape the chain elongation reactor microbiota (9)	Bioreactor	136	50.0	100.0	27.2	87.5	0.0022	0.7966	0.0189	0.0112
**PRJNA552606**	The occurrence and ecology of microbial chain elongation of carboxylates in soils (10)	Enrichments from soils	54	40.7	66.7	35.2	25.9	0.0404	0.1758	0.2094	0.0852
**PRJNA556976**	Enrichment and characterisation of ethanol chain elongating communities from natural and engineered environments (11)	Enrichments from anaerobic digestors and feces	66	16.7	93.9	33.3	21.2	0.1552	40.5209	0.5081	0.0384
**PRJNA808231**	Lactate and ethanol chain elongation in presence of lactose (12)	Bioreactor	19	89.5	10.5	68.4	52.6	0.3521	0.0936	0.0290	0.0132
**PRJNA973099/ PRJNA954053**	Lake Washington wetland (This study)	Wetland	5	60.0	40.0	100.0	20.0	0.0045	0.0258	0.0028	0.0092
**PRJNA575743**	Microbial diversity in boreal wetlands (13)	Wetland	3	0.0	0.0	33.3	33.3	0.0000	0.0000	0.0027	0.0133
**PRJNA555827**	Karst wetland metagenome (14)	Wetland	28	3.6	0.0	3.6	3.6	0.0021	0.0000	0.0022	0.0021
**PRJNA731194, 195,197,198,200 - 218,221,224-227,231,232**	Salt pond soil microbial communities from wetland under restoration in South San Francisco, CA, USA (15)	Wetland	30	0.0	0.0	0.0	0.0	0.0000	0.0000	0.0000	0.0000
**PRJNA338276**	Old Woman Creek wetland (16)	Wetland	66	1.5	0.0	10.6	10.6	0.0141	0.0000	0.0045	0.0110

S.3. References

1. Candry P, Radic L, Favere J, Carvajal-Arroyo JM, Rabaey K, Ganigué R. Mildly acidic pH selects for chain elongation to caproic acid over alternative pathways during lactic acid fermentation. Water Res. 2020;186(116396):1–9.
2. Parada AE, Needham DM, Fuhrman JA. Every base matters: Assessing small subunit rRNA primers for marine microbiomes with mock communities, time series and global field samples. Environ Microbiol. 2016 May 1;18(5):1403–14.
3. Edgar RC, Bateman A. Search and clustering orders of magnitude faster than BLAST. Bioinformatics. 2010 Oct 1;26(19):2460–1.
4. Edgar RC. UNOISE2: improved error-correction for Illumina 16S and ITS amplicon sequencing. bioRxiv. 2016 Oct 15;081257.
5. Cole JR, Wang Q, Fish JA, Chai B, McGarrell DM, Sun Y, et al. Ribosomal Database Project: data and tools for high throughput rRNA analysis. Nucleic Acids Res. 2014 Jan 1;42(D1):D633–42.
6. Kumar S, Stecher G, Li M, Knyaz C, Tamura K. MEGA X: Molecular evolutionary genetics analysis across computing platforms. Mol Biol Evol. 2018;35(6):1547–9.
7. Tamura K, Nei M. Estimation of the Number of Nucleotide Substitutions in the Control Region of Mitochondrial DNA in Humans and. Mol Biol Evol. 1993;10(3):512–26.
8. Letunic I, Bork P. Interactive Tree of Life (iTOL) v4: Recent updates and new developments. Nucleic Acids Res. 2019;47(W1):256–9.
9. Liu B, Sträuber H, Centler F, Harms H, da Rocha UN, Kleinsteuber S. Functional Redundancy Secures Resilience of Chain Elongation Communities upon pH Shifts in Closed Bioreactor Ecosystems. Environ Sci Technol. 2022;
10. Joshi S, Robles A, Aguiar S, Delgado AG. The occurrence and ecology of microbial chain elongation of carboxylates in soils. ISME J. 2021;15:1907–18.

11. Candry P, Huang S, Carvajal-arroyo JM, Rabaey K, Ganigue R. Enrichment and characterisation of ethanol chain elongating communities from natural and engineered environments. Sci Rep. 2020;10(3682):1–10.
12. Duber A, Zagrodnik R, Gutowska N, Łężyk M, Oleskowicz-Popiel P. Lactate and Ethanol Chain Elongation in the Presence of Lactose: Insight into Product Selectivity and Microbiome Composition. ACS Sustain Chem Eng. 2022 Mar 21;10(11):3407–16.
13. Schaefer JK, Kronberg RM, Björn E, Skyllberg U. Anaerobic guilds responsible for mercury methylation in boreal wetlands of varied trophic status serving as either a methylmercury source or sink. Environ Microbiol. 2020 Sep 1;22(9):3685–99.
14. Yan Z, Li W, Shen T, Wang X, Zhu M, Yu L. Aquatic microalgal and bacterial communities in a karst wetland watershed. J Hydrol (Amst). 2020 Dec;591:125573.
15. Zhou J, Theroux SM, Bueno de Mesquita CP, Hartman WH, Tian Y, Tringe SG. Microbial drivers of methane emissions from unrestored industrial salt ponds. The ISME Journal 2021 16:1 [Internet]. 2021 Jul 28 [cited 2023 Apr 19];16(1):284–95. Available from: https://www.nature.com/articles/s41396-021-01067-w
16. Angle JC, Morin TH, Solden LM, Narrowe AB, Smith GJ, Borton MA, et al. Methanogenesis in oxygenated soils is a substantial fraction of wetland methane emissions. Nat Commun [Internet]. 2017;8(1):1–9. Available from: http://dx.doi.org/10.1038/s41467-017-01753-4
